# Translateral and Transtibial Techniques Provide Similar Patient Outcomes in Arthroscopic Anterior Cruciate Ligament Reconstruction

**DOI:** 10.1002/ars2.70019

**Published:** 2026-06-08

**Authors:** Joel Yat Seng Wong, Christian Hwee Yee Heng, Andrew Hwee Chye Tan

**Affiliations:** ^1^ Department of Orthopaedic Surgery Singapore General Hospital Singapore Singapore

## Abstract

**Purpose:**

To compare the outcomes of the transtibial technique (TT) and the translateral technique (TLL) for anterior cruciate ligament (ACL) reconstruction in an Asian population.

**Methods:**

Patients who underwent either TT or TLL for ACL reconstruction between 2019 and 2021 were identified. Inclusion criteria were skeletal maturity, a magnetic resonance imaging–diagnosed ACL tear, completion of at least 2 of 3 postoperative assessments, and a minimum follow‐up of 24 months. Data collected include patient demographics, patient‐reported outcome measure scores (Tegner activity scale and Lysholm score), objective clinical outcome scores (KT‐1000), and medical records review to identify complications.

**Results:**

The average follow‐up time was 15.2 months (range: 6 to 24 months) in TT versus 17.5 months (range: 6 to 24 months) in TLL, *P* value = .622. Recovery of quadriceps function in a cohort of 134 patients was identified as the only significant difference in postoperative functional outcomes between the TT and TLL groups. At 12 months, the extension range of motion in TLL was statistically better than TT (TT: 1.1 ± 3.4 [–2.2, 4.4] vs TLL: –0.9 ± 2.9 [–3.7, 2.0], *P* = .005). KT‐1000 measurement, Tegner activity scale, and Lysholm score were not statistically significant between the TT and TLL groups at 24 months. The follow‐up rate (n [%]) between the TT and TLL groups at 12 months was 59 (78.7%) and 50 (84.7%), respectively, and at 24 months was 55 (77.3%) and 47 (79.7%), respectively.

**Conclusions:**

In this Asian cohort, transtibial and translateral ACL reconstruction showed similar short‐term outcomes. Small, transient differences in extension recovery favoring TLL did not persist at 24 months.

**Level of Evidence:**

Level III, retrospective comparative study.

## Graphical abstract

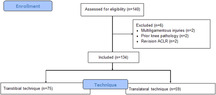

The anterior cruciate ligament (ACL) is the most common injured ligament in the knee,^1^ and ACL reconstruction (ACLR) has become one of the most common orthopaedic surgery procedures.^2^ Although ACLR is a prevalent procedure, there has yet to be consensus on which surgical technique is superior.^2^ Graft positioning is paramount in conferring rotational stability to ACLR.^3^, ^4^ The choice of technique influences graft positioning. The 2 techniques that are commonly used in clinical practice are the transtibial technique (TT) and translateral technique (TLL). TT exists as a classical, conventional technique that involves transtibial drilling to create the femoral tunnel toward the roof of the intercondylar notch or deep posteriorly along the Blumensaat's line or both.^5^ The resultant outcome of TT is a vertically orientated graft, owing to the increased tendency of creating a nonanatomic graft position whereby the graft is placed anterior on the femur and posterior onto the tibia.^5^ Vertically oriented ACLR grafts are reported to result in postoperative failure to restore normal keen kinematics with rotational laxity, rotational knee instability, and graft failure.^6^, ^7^, ^8^, ^9^, ^10^ To address the shortcomings of TT, TLL was developed to create a more anatomic graft position through independent anatomic femoral tunnel placement and creation of retrograde sockets at the discretion of the surgeon. TLL can achieve good clinical and functional outcomes with low complication and failure rates.^11^, ^12^, ^13^

This study represents the evolution of a previous study aimed at investigating the outcomes of ACLR between fixed and adjustable loop devices.^14^ Several studies have focused on biomechanical properties of the most common femoral and tibial fixation implants,^15^, ^16^, ^17^, ^18^, ^19^, ^20^, ^21^ predominantly in Western populations. Differences in bone structure between Asians and Westerners have been described in the literature.^22^, ^23^, ^24^ Thus, the resultant difference in anthropometry and femoral notch morphology in Asians compared with Westerners^25^, ^26^, ^27^, ^28^ leads to theoretical biomechanical differences in movement and variation in strain loads on the ACLR graft. Excessive graft strain predisposes to postoperative mechanical failure^29^, ^30^ and poor clinical outcomes. This study focuses on the Asian population because the country in which our institution resides is predominantly Asian. The purpose of this study was to compare the outcomes of the TT and the TLL for ACLR in an Asian population. We hypothesized that the TLL would yield clinically superior outcomes over the TT.

## METHODS

### Time Frame of Study and Evaluation Methods

**FIGURE 1:**
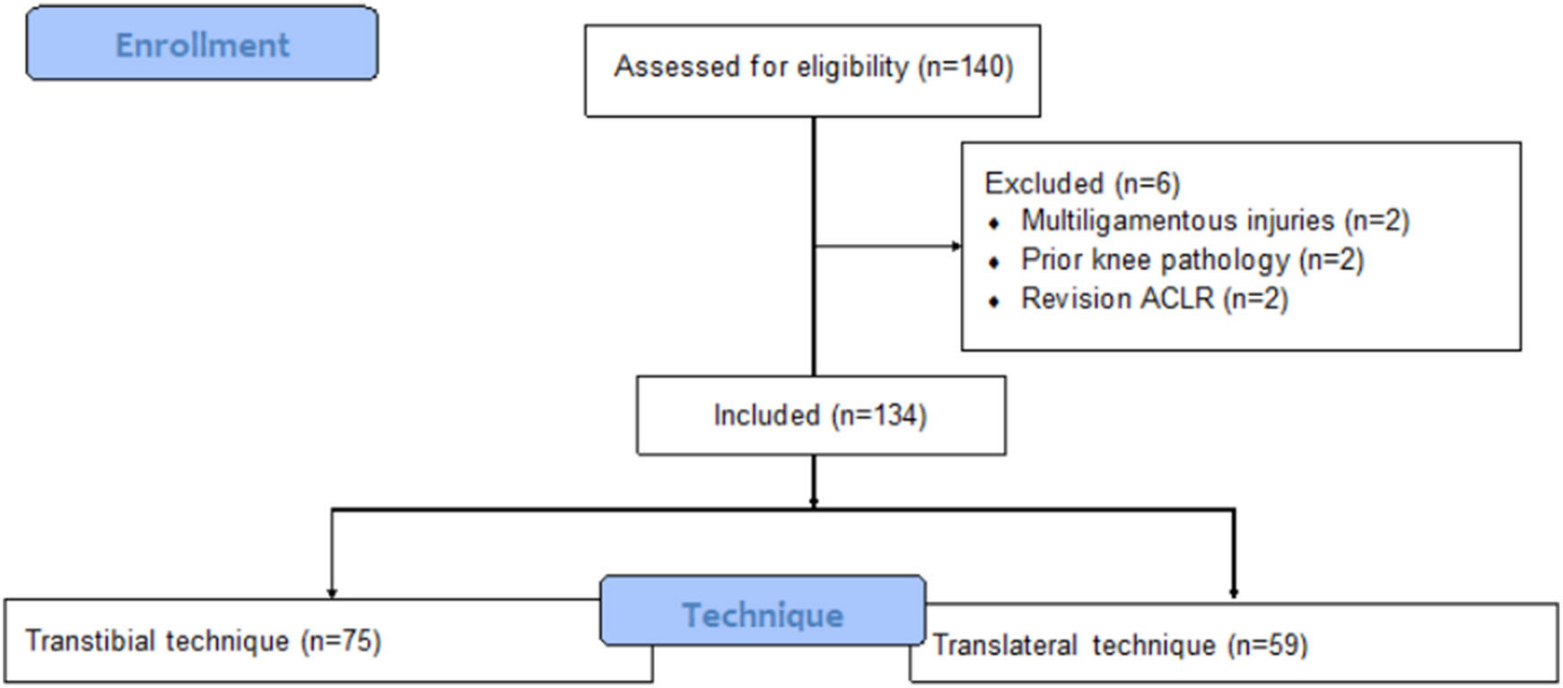
Flowchart of patient inclusion.

Patients who underwent ACLR between 2019 and 2021 were investigated to compare 2 different surgical techniques of ACLR with both patient‐reported outcome measures (PROMs) and range of motion (ROM) at least 2 years after ACLR. Inclusion criteria were skeletal maturity, a magnetic resonance imaging–diagnosed ACL tear, and completion of at least 2 of 3 postoperative assessments. Exclusion criteria were multiligamentous injuries, prior knee pathology, and revision ACLR. Patients with concomitant injuries such as meniscus tear and chondral injury were not excluded. Patients were segregated into 2 groups, TT, which used Zimmer Biomet ToggleLoc with ZipLoop (TL) as an adjustable‐loop device, and TLL, which used Arthrex GraftLink (GL) with also adjustable loop devices at both ends of the graft. Clinical assessment was performed preoperatively and postoperatively by the operating surgeon (A.H.C.T.) at regular intervals of 6, 12, and 24 months. All patients were registered into an ACL injury database; all patients in the database would have PROMs and clinical measures (such as KT‐1000 scores) recorded in the database. KT‐1000 knee ligament arthrometer (MEDmetric) was used to test for anterior translational instability^20^, ^21^ at 30 lb. force, of which side‐to‐side difference was subsequently calculated; 30 lb. was used as a standardized measurement in our institution. Physiotherapists at our institution's Orthopaedic Diagnostic Centre administered Questionnaires and scales to patients to assess clinical and functional knee outcomes: Tegner activity scale (TAS) was used to measure physical performance (1‐10); Lysholm score (LS) was used for subjective evaluation. Data collected include patient demographics, PROM scores (TAS and LS), objective clinical outcome scores (KT‐1000), and medical record review to identify complications. Pivot‐shift grading or quantitative rotation testing was not included as our center had no objective method to collect these data (Figure 1).

### Specific Indication for Surgery

Indications for surgery were patients who expressed dissatisfaction in their lifestyle after experiencing an ACL injury. Such individuals are motivated with high activity and functional demands.

### Surgical Technique

All ACLRs were performed by a single fellowship‐trained orthopaedic sports surgeon with 2 decades of experience (A.H.C.T.). Due to personal preference, the surgeon's preferred technique of ACLR evolved from TT to TLL with the passage of time. The surgeon used TT only from 2019 to mid‐2020 and used TLL only from mid‐2020 to 2021. The graft fixation adjustable‐loop device devices used were either TL or GL. Other concomitant meniscus and cartilage pathologies were also treated intraoperatively. Suspensory fixation was performed on both tibial and femoral sides. For TT, both gracilis (G) and semitendinosus (ST) were harvested and doubled (“doubled G/ST”). For TLL, only ST was harvested and quadrupled (“quadrupled ST”). Regardless of technique, grafts were fashioned to be at least 7.5 mm in diameter. After insertion of the graft, the graft was tightened until taut and cycled. In TT, creation of the femoral tunnel was toward the roof of the intercondylar notch, resulting in a more vertically oriented graft placement. In TLL, creation of the femoral tunnel was independent of the tibial tunnel, resulting in a more horizontal graft positioning.

### Postoperative Rehabilitation

Patients were prescribed a postoperative knee brace, which provided varus/valgus restraint, and ROM was allowed until 90 degrees. Crutches were used for the first 4 weeks. Full weightbearing was allowed after 2 weeks if no meniscus repair was performed and after 6 weeks if meniscus repair was performed. Physiotherapy commenced on postoperative day 1. Return to sports was not recommended earlier than 9 to 12 months postoperatively. Patients received standardized rehabilitation protocol that was identical between groups under the supervision of a sports physiotherapist. Immediate postoperative rehabilitation protocols were aimed at protecting the graft, regaining full active extension, and re‐establishing quadriceps control. Patients were educated to keep their knee straight and avoid resting with a towel placed under the knee, and not to pivot on the surgical side. The intermediate postoperative period focused on maintaining full flexion and normalizing gait. Stretching exercises taught to patient were prone and standing quadriceps stretch and kneeling hip flexor stretch. Strengthening exercises include prone and standing hamstring curls. The transitional period placed emphasis on maintaining full ROM with sport‐specific training in the sagittal plane.

### Statistical Analysis

Statistical analysis was performed using Microsoft Excel 2019. A Kolmogorov‐Smirnov test was used to verify that the data follow a normal distribution. Normally distributed continuous variables were presented as descriptive data in the form of mean and standard deviation. Categorical variables were presented as sample size and percentages.

Subjects were grouped according to the ACLR surgical technique used. An unpaired 2‐sample Student *t* test was used to compare each group with one another.

Comparison of primary outcomes included overall means for the KT‐1000 test, Tegner, LS, and knee ROM. A confidence interval of 95% was considered statistically significant. With a power of 80% and a type I error rate of 5%, we calculated that we needed 30 patients for each group (60 patients in total).^31^ Our sample size was larger than the minimum required. Clinical significance was evaluated using minimal clinically important differences (MCIDs), the smallest change in outcome that a patient is able to perceive and appreciate.^32^, ^33^ The utility of the MCID is to determine the efficacy of a treatment by focusing on the clinical value rather than on statistical significance.^34^ We consider a clinically significant difference as a change in the mean outcome score that exceeded a previously determined MCID for that particular outcome (*P* < .05). We determined maximal medical improvement by identifying the latest period where the change in singular outcome score did not exceed the MCID. In our study, we defined the MCID in alignment with the prevailing literature, where MCID for the LS was determined to be 10.^35^, ^36^

## RESULTS

All data were normally distributed (Kolmogorov‐Smirnov test, *P* > .05) and between‐group variances were equal (Levene's test, *P* > .05).

### Demographics

A total of 134 patients were included in the study, consisting of 75 and 59 patients from TT and TLL, respectively (Table 1). The average follow‐up time was 15.2 months (range: 6 to 24 months) in TT versus 17.5 months (range: 6 to 24 months) in TLL, *P* value = .622. No statistically significance difference was found between the 2 groups. Analysis and comparison based on sex were not performed because the differences between the male and female sex pertaining to the number of participants, height, weight, and body mass index were not statistically significant (Table 1).

**TABLE 1:** Demographics, Main Group

	TT (n = 75)	TLL (n = 59)	*P* Value
Age, yr, mean ± SD	29.7 ± 8.5	31.0 ± 10.0	.414
Male: Female, n (%)	50 (67.7%): 25 (33.3%)	42 (71.2%): 17 (28.8%)	.579
*Ethnicity, n (%)*			
Southeast Asian	67 (89.3%)	54 (91.5)	.110
Non–Southeast Asian	8 (10.7%)	5 (8.5%)	.262
Mean time from first physiotherapy assessment to surgery, mo, mean ± SD	0.5 ± 0.6	0.4 ± 0.4	.474
Height, cm, mean ± SD	167.8 ± 10.6	170.2 ± 6.6	.135
Weight, kg, mean ± SD	72.7 ± 19.3	75.1 ± 14.3	.578
BMI, mean ± SD	27.2 ± 20.0	25.9 ± 4.9	.452
*Follow‐up rate, n (%)*			
6 mo	66 (88.0%)	52 (88.1%)	.133
12 mo	59 (78.7%)	50 (84.7%)	.358
24 mo	55 (73.3%)	47 (79.7%)	.552
Average follow‐up, mo	15.2	17.5	.622

BMI, body mass index; mo, month; SD, standard deviation; TLL, translateral technique; TT, transtibial technique; yr, year.

### ROM

Significantly higher flexion ROM was observed preoperatively for the patients in TLL compared with those in the TT (TT: 127.3 ± 23.6 vs TLL: 134.5 ± 12.9, *P* = .030) (Table 2A). For extension ROM, a lower numerical value represented better extension (i.e., closer to zero degrees), hence greater recovery of quadriceps strength.^22^ At 12 months, significantly better extension was observed for patients in TLL compared with TL (TT: 1.1 ± 3.4 vs TLL: –0.9 ± 2.9, *P* = .005) (Table 2A). This meant that TLL was able to allow better postoperative extension at 12 months compared with TL. All groups had statistically significant postoperative improvement in knee flexion when compared with baseline (Table 2B).

**TABLE 2A:** Comparison of Knee ROM Between Transtibial and Translateral Groups

	Knee ROM, Degrees					
	Extension			Flexion		
	TT (n = 75)	TLL (n = 59)	*P* Value	TT (n = 75)	TLL (n = 59)	*P* Value
Preoperative	1.7 ± 6.2 (–4.4, 7.8)	2.9 ± 6.4 (–3.4, 9.2)	.279	127.3 ± 23.6 (103.6, 150.8)	134.5 ± 12.9 (121.5, 147.4)	.030*
6 month	–0.3 ± 3.9 (–4.1, 3.5)	–0.4 ± 3.2 (–3.5, 2.7)	.864	136.6 ± 9.4 (127.1, 146.0)	137.4 ± 10.2 (127.1, 147.5)	.661
12 month	1.1 ± 3.4 (–2.2, 4.4)	–0.9 ± 2.9 (–3.7, 2.0)	.005*	137.5 ± 10.0 (127.4, 147.4)	137.3 ± 11.2 (126.0, 148.4)	.946
24 month	–1.3 ± 3.8 (–5.0, 2.4)	–2.4 ± 3.1 (–5.4, 0.6)	.196	138.0 ± 7.9 (130.1, 145.8)	138.6 ± 9.3 (129.2, 147.8)	.799

ROM, range of motion; TLL, translateral technique; TT, transtibial technique.

* Significant *P* value < .05.

**TABLE 2B:** Comparison of Preoperative and Postoperative Knee ROM

	Knee ROM, Degrees			
	Extension		Flexion	
	*P* Value		*P* Value	
	TT	TLL	TT	TLL
Preoperative vs 6 month	.020*	.001*	.245*	.012*
Preoperative vs 12 month	.045*	.015*	.007*	.008*
Preoperative vs 24 month	.012*	.038*	.033*	.002*

ROM, range of motion; TLL, translateral technique; TT, transtibial technique.

* Significant *P* value < .05.

### Stability (KT‐1000 Testing)

Preoperatively, all patients had positive Lachman's and pivot‐shift tests. The mean preoperative manual KT‐1000 arthrometer side‐to‐side laxity difference ranged from 10.6 to 11.9 mm. No statistical differences were found among the 2 groups for mean and side‐to‐side difference (Table 3A). All groups had statistically significant postoperative improvement in KT‐1000 measurement compared with baseline (Table 3B).

**TABLE 3A:** Comparison of Clinical Laxity Evaluation and Subjective Outcome Improvement Between Transtibial and Translateral Technique

	KT‐1000 Measurement, mm					
	Mean			Side‐to‐Side Difference		
	TT (n = 75)	TLL (n = 59)	*P* Value	TT (n = 75)	TLL (n = 59)	*P* Value
Preoperative	10.6 ± 3.5 (7.0, 14.0)	11.9 ± 4.3 (7.5, 16.1)	.069	3.4 ± 3.0 (0.3, 6.3)	3.9 ± 3.7 (0.2, 7.5)	.434
6 month	9.6 ± 3.1 (6.4, 12,6)	10.2 ± 3.2 (7.0, 13.3)	.381	2.0 ± 2.5 (0.5, 3.5)	1.6 ± 3.3 (‐1.7, 4.9)	.570
12 month	10.4 ± 3.1 (7.3, 13.4)	9.8 ± 3.3 (6.4, 13.0)	.427	2.0 ± 2.2 (1.0, 3.0)	2.2 ± 2.8 (1.8, 2.6)	.749
24 month	10.2 ± 2.3 (7.8, 12.5)	10.5 ± 2.8 (7.6, 13.2)	.674	2.2 ± 2.0 (0.1, 4.1)	1.8 ± 2.8 (1.4, 2,2)	.641

TLL, translateral technique; TT, transtibial technique.

**TABLE 3B:** Comparison of Preoperative and Postoperative Clinical Laxity Evaluation and Subjective Outcome Improvement

	KT‐1000 Measurement, mm			
	Mean		Side‐to‐side Difference	
	*P* Value		*P* Value	
	TT	TLL	TT	TLL
Preoperative vs 6 month	.009*	.021*	.014*	.008*
Preoperative vs 12 month	.002*	.042*	.011*	.003*
Preoperative vs 24 month	.040*	.033*	.021*	.029*

TLL, translateral technique; TT, transtibial technique.

* Significant *P* value < .05.

### Subjective Evaluation

#### LS

The difference in the LS was not statistically significant (Table 4A). All groups had statistically significant postoperative improvement in LS (Table 4B).

**TABLE 4A:** Comparison of Patient Reported Outcomes Between Transtibial and Translateral Technique

	Lysholm Score			Tegner Activity Scale		
	TT (n = 75)	TLL (n = 59)	*P* Value	TT (n = 75)	TLL (n = 59)	*P* Value
Preoperative	63.2 ± 4.3 (58.8, 67.4)	64.4 ± 21.1 (43.2, 85.4)	.741	6.5 ± 1.8 (4.6, 8.2)	6.5 ± 1.4 (5.1, 7.8)	.938
6 month	87.2 ± 11.5 (75.6, 98.6)	88.0 ± 11.7 (76.2, 99.6)	.760	4.0 ± 1.6 (2.4, 5.5)	4.0 ± 1.5 (2.4, 5.4)	.927
12 month	90.4 ± 8.4 (81.9, 98.7)	91.3 ± 11.5 (79.7, 102.7)	.738	5.0 ± 1.7 (3.3, 6.7)	5.1 ± 1.6 (3.4, 6.6)	.901
24 month	92.8 ± 6.9 (85.8, 99.6)	93.8 ± 9.6 (84.1, 103.3)	.740	5.4 ± 2.0 (3.3, 7.3)	5.8 ± 1.2 (4.5, 6.9)	.455

TLL, translateral technique; TT, transtibial technique.

**TABLE 4B:** Comparison of Preoperative and Postoperative Patient‐Reported Outcomes

	Lysholm Score		Tegner Activity Scale	
	*P* Value		*P* Value	
	TT	TLL	TT	TLL
Preoperative vs 6 month	.542	.232	.114	.387
Preoperative vs 12 month	.629	.459	.478	.645
Preoperative vs 24 month	.358	.891	.764	.523

TLL, translateral technique; TT, transtibial technique.

#### TAS

The difference in the TAS was not statistically significant (Table 4A). All groups had statistically significant postoperative improvement in TAS (Table 4B).

#### Complications and Additional Procedures

At the 24‐month follow‐up, there were no revision ACLRs and no postoperative surgical site infections.

#### MCID

Both TT and TLL groups had more than 80% of subjects that met or exceeded the MCID threshold values.

## DISCUSSION

Our study found that recovery of quadriceps function (extension ROM) was the only significant difference in postoperative functional outcomes between TT and TLL (TT [TL]: 1.1 ± 3.4 vs TLL [GL]: –0.9 ± 2.9, *P* = .005) (Table 2A). There was a lost‐to‐follow‐up rate of 26.7% and 20.3% in TT and TLL, respectively, although this did not reach statistical significance (Table 1).

Regardless of surgical technique, grafts were fashioned to be at least 7.5 mm in diameter. For TT, both gracilis (G) and semitendinosus (ST) were harvested and doubled (“doubled G/ST”). For TLL, only ST was harvested and quadrupled (“quadrupled ST”). Quadrupled ST graft allows only the semitendinosus tendon to be used because the fashioned graft is appropriately wide.^37^ Inagaki et al. found no significant differences between the 2 graft fashioning techniques (quadrupled ST graft vs doubled G/ST graft) concerning knee stability and postoperative outcome.^38^ Although differences in graft preparation are unlikely to influence the results, it may still be a potential confounder because the resultant differences in graft bundle size, strength, and healing characteristics^39^, ^40^ could overshadow technique‐related effects.

The key differentiating aspect of our study is its focus on a predominantly Asian population. Much literature has investigated the biomechanical properties of common femoral and tibial fixation implants^15^, ^16^, ^17^, ^18^, ^19^, ^20^, ^21^ in predominantly Western populations. However, the differences in anthropometry and femoral notch morphology in Asians compared with Westerners^25^, ^26^, ^27^, ^28^ that arise from differences in bone structure between Asians and Westerners^22^, ^23^, ^24^ will lead to theoretical biomechanical differences in movement and variation in strain loads on the ACLR graft. Excessive graft strain increases the likelihood of postoperative mechanical failure^29^, ^30^ and poor clinical outcomes.

Creation of the femoral tunnel is a crucial aspect of ACLR. The key difference between TT and TLL lies in the approach to tunnel placement and hence difference in graft position. Prior to the introduction of TLL in 2012,^41^ the TT technique was the technique of choice for most surgeons, in which creation of the femoral tunnel is performed through the tibial tunnel.^42^ Supporters of the TT cite multiple advantages, including performance ease, ability to reach the center of the native ACL footprint, and avoiding the need for special equipment.^43^ Although positive early clinical outcomes have been observed with TT, the dictation of femoral socket placement by the tibial tunnel results in a relatively nonanatomic vertical position of the ACL graft.^41^, ^44^, ^45^ Subsequent functional sequelae of nonanatomic ACL graft positioning include failure to restore normal knee kinematics,^7^, ^45^ resulting in increased propensity for disproportionate stress exerted on the graft. This, in turn, increases the likelihood of graft failure, femoral posterior wall rupture,^11^, ^46^, ^47^, ^48^ and potential early‐onset knee osteoarthritis.^49^ Others have also observed that the TT shows higher risk of graft impingement, rotational instability, and graft attenuation^10^, ^50^ due to intrinsic vertical graft positioning.

In response to the nonanatomic TT technique, ACLR evolved to “anatomic” or “footprint” techniques, one of which is the TLL technique.^41^ Anatomic femoral tunnel creation in TLL is achieved through femoral tunnel creation that is independent of the tibial tunnel.^11^, ^51^, ^52^, ^53^, ^54^ This femoral tunnel versatility in TLL is a cornerstone advantage of TLL over TT, allowing surgeons to exercise discretion on where to place the femoral tunnel to achieve optimal trajectory based on interpretation of the femoral structure and expertise.^55^ The horizontal graft positioning in TLL confers rotational stability, with biochemical and clinical studies showing improved stability that can be attributed to anatomical positioning of the femoral tunnel in the TLL^11^, ^56^, ^57^ compared with nonanatomic reconstruction. Our study found no detectable differences in in KT‐1000 Testing (translational stability) between TT and TLL (Table 3A). The clinical outcome scores suggest that a more horizontal graft positioning offered in TLL does not appear to confer greater translational stability.

### Limitations

This study has limitations to consider. First, the short follow‐up period of 2 years may not be long enough to assess the durability of the repair. The small sample size creates a risk for a type II error. Second, while suspensory graft fixation is used in both TT and TLL, only the femoral end of the graft is adjustable in TT technique. In contrast, both ends of the graft are adjustable in TLL. This may be a confounder. Third, to standardize results, our study did not include physical examination findings such as the pivot shift test, which may be subjective depending on the examiner. Thus, the rotational stability of the graft was not objectively measured. Next, the lack of regression or propensity matching for group differences at baseline (meniscal injury status, ROM) remains uncontrolled and may introduce confounding variables and selection bias leading to biased estimates of treatment effects.^58^ Finally, the loss to follow‐up (20%‐27%) presents an attrition bias and could meaningfully bias results.

## CONCLUSIONS

In this Asian cohort, transtibial and translateral ACL reconstruction showed similar short‐term outcomes. Small, transient differences in extension recovery favoring TLL did not persist at 24 months.

## DISCLOSURES

The authors (J.Y.S.W., C.H.Y.H., A.H.C.T.) declare that they have no known competing financial interests or personal relationships that could have appeared to influence the work reported in this paper.
